# A quantitative and qualitative review of the effects of testosterone on the function and structure of the human social-emotional brain

**DOI:** 10.1007/s11011-015-9692-y

**Published:** 2015-06-16

**Authors:** Sarah J. Heany, Jack van Honk, Dan J. Stein, Samantha J. Brooks

**Affiliations:** Department of Psychiatry and Mental Health, University of Cape Town, Groote Schuur Hospital, J2, Anzio road, Observatory, Cape Town South Africa; Department of Psychology, Utrecht University, Utrecht, The Netherlands; Institute of Infectious Diseases and Molecular Medicine, University of Cape Town, Cape Town, South Africa; Department of Neuroscience, Uppsala University, Uppsala, Sweden

**Keywords:** Testosterone, ALE, VBM, fMRI, Amygdala, Hippocampus

## Abstract

Social and affective research in humans is increasingly using functional and structural neuroimaging techniques to aid the understanding of how hormones, such as testosterone, modulate a wide range of psychological processes. We conducted a meta-analysis of functional magnetic resonance imaging (fMRI) studies of testosterone administration, and of fMRI studies that measured endogenous levels of the hormone, in relation to social and affective stimuli. Furthermore, we conducted a review of structural MRI i.e. voxel based morphometry (VBM) studies which considered brain volume in relation to testosterone levels in adults and in children. In the included testosterone administration fMRI studies, which consisted of female samples only, bilateral amygdala/parahippocampal regions as well as the right caudate were significantly activated by social-affective stimuli in the testosterone condition. In the studies considering endogenous levels of testosterone, stimuli-invoked activations relating to testosterone levels were noted in the bilateral amygdala/parahippocampal regions and the brainstem. When the endogenous testosterone studies were split by sex, the significant activation of the brain stem was seen in the female samples only. Significant stimuli-invoked deactivations relating to endogenous testosterone levels were also seen in the right and left amygdala/parahippocampal regions studies. The findings of the VBM studies were less consistent. In adults larger volumes in the limbic and temporal regions were associated with higher endogenous testosterone. In children, boys showed a positive correlation between testosterone and brain volume in many regions, including the amygdala, as well as global grey matter volume, while girls showed a neutral or negative association between testosterone levels and many brain volumes. In conclusion, amygdalar and parahippocampal regions appear to be key target regions for the acute actions of testosterone in response to social and affective stimuli, while neurodevelopmentally the volumes of a broader network of brain structures are associated with testosterone levels in a sexually dimorphic manner.

**Electronic supplementary material:**

The online version of this article (doi:10.1007/s11011-015-9692-y) contains supplementary material, which is available to authorized users.

## Introduction

Testosterone has been associated with aggression, violence, and sexually motivated behavior (Dabbs et al. 1987; Isidori et al. 2005; Montoya et al. 2012), perhaps by reducing impulse control (Struber et al. 2008). However, a growing number of neuroimaging and single-dose administration studies suggest that testosterone has a more nuanced role in social and emotional behaviors, and especially promotes dominance behavior (Eisenegger et al. 2010; van Honk et al. 2012). Crucially, hormones interact with social environments, and both the affective and strategic behaviors they facilitate can only be understood in terms of hormone-environment interactions. However, although there is increasing research on the topic (Bos et al. 2012b), the exact neural mechanisms by which testosterone acts on the brain remain under debate.

A systematic review of the literature of structural MRI in relation to testosterone levels may shed light on neurodevelopmental effects of the hormone. Additionally, a meta-analysis of social and affective neuroimaging, which considers both studies using endogenous and exogenous testosterone, may provide insights into how testosterone acts in the brain to influence our social and emotional behavior, and whether activity of brain regions differs in relation to endogenous and exogenous variations in testosterone.

In a recent qualitative review of clinical and preclinical neuroimaging studies it is suggested that testosterone plays a role in the pathogenesis of psychiatric disorders such as depression (Höfer et al. 2013), on the basis of hormonal interactions in the limbic system, but there have not been any quantitative reviews of the effects of endogenous and exogenous testosterone on the social-emotional brain.

Here we conduct a quantitative meta-analysis using Activation Likelihood Estimation (ALE) in fMRI studies that have examined the neural activations and deactivations related to endogenous levels of testosterone, as well as the neural responses to exogenous doses, during social emotional processing. This is in line with other ALE studies conducted by authors in our group (Brooks et al. 2012; Hattingh et al. 2013). Additionally, we present a qualitative review of structural brain imaging studies in which endogenous testosterone was the independent variable.

## Methods

### Searching procedure

Online academic search engines of PubMed, Sciencedirect, Ovid, EBSCOHost, and Google Scholar were searched. Additional hand searches were performed from reference lists of relevant articles. Articles from all years, until October 2013 were accepted. The following search parameters were used: “testosterone fMRI” (*n* = 452), “eeg testosterone” (*n* = 129), “emg testosterone” (*n* = 74), plethysmography testosterone” (*n* = 21), “trust testosterone” (*n* = 290), “empathy testosterone” (*n* = 20), anxiety testosterone” (*n* = 448), “[(testosterone MRI) - (testosterone fMRI)] (*n* = 88), “testosterone VBM” (*n* = 5). A total of 1527 papers were found by these means. Based on the findings of this initial literature search, it was decided that only studies using MRI methods (fMRI and VBM analyses) would be used in this review.

### Inclusion and exclusion criteria

Studies were selected according to set inclusion and exclusion criteria. To be included in the analyses, studies needed to satisfy the following criteria: a) fMRI studies providing Talairach or MNI coordinates of significant regions. Other brain scanning modalities, such as positron emission tomography (PET) or diffusion tensor imaging (DTI), were excluded b) Only studies with healthy adult participants were included. c) Studies which contain a direct measure of testosterone via sampling either saliva or blood d) Original articles published in English. e) Studies which include a sample size of at least 10 f. Published in a peer-reviewed journal g) Studies reporting whole brain (WB) or region of interest (ROI) coordinates.

### Identification, screening, eligibility

The study selection process is detailed in a Preferred Reporting Items for Systematic Reviews and meta-Analyses (PRISMA) supplementary figure F. During an initial screening, 1395 of the original 1527 were excluded. The 132 remaining articles underwent a secondary screen; 80 included behavioral tasks only, 5 fMRI studies used cognitive tasks, 14 were electroencephalography (EEG) or electromyography (EMG) studies, 2 looked at fMRI region coupling rather than activations. This left 31 studies to be included in this meta-analysis. Six of these were appropriate fMRI testosterone administration (exogenous) studies, 9 were fMRI studies assessing endogenous testosterone, and 16 were voxel based morphometry (VBM) papers, of which 10 used child samples.

Many of the remaining exogenous and endogenous papers provided ROI coordinates as their significant findings. These were included in the final set of papers, and in the analyses. While we are aware that including ROI analyses might over-inflate the results of our ALE analysis (Eickhoff et al. 2012), we included all appropriate ROI findings in this preliminary analysis to increase the number of studies. However, in future, it will be more beneficial to include only whole-brain analyses once more studies have been conducted.

### Exogenous and endogenous testosterone fMRI studies

The exogenous studies we included in the meta-analysis all used similar administration paradigms; all administered either 0.5 mg or 0.9 mg of testosterone sublingually or intranasally to exclusively female samples, and were placebo controlled. In order to keep the population homogenous, only healthy premenopausal female samples were included, as this is what most of the studies used. There were not sufficient deactivation foci to run a separate deactivation analysis, so only activated regions were included in our analyses of administration studies. Conversely, for the endogenous papers we were able to collect data for activations and deactivations, with some studies reporting both. Using the endogenous studies, separate analyses were run for activated and deactivated regions. Further, given that these endogenous studies either had male or female participants, or both, we were able to run separate analyses for female activations and male activations. Those studies that had a mixed sample of males and females were excluded from the secondary analyses where we examined gender separately. There were not sufficient studies to run analyses looking at deactivations separately for men and women. It must be noted that sex differences were not directly examined in this meta-analysis, but rather the differences within sex groups with testosterone used as a contrast (exogenous) or continuous (endogenous) variable. For the overall analysis of the effects of exogenous testosterone on brain function, there were 6 included studies, whereas for the analyses examining the effects of endogenous testosterone on brain function, in total there were 9 studies, 5 for males only, 1 for females only, and 3 with a combined sample.

### Tasks used in the included fMRI studies

All the placebo controlled testosterone administration functional neuroimaging studies included healthy females only. This is due to there being an established administration paradigm for a female appropriate dose, but there is no such equivalent paradigm validated for male participants (Tuiten et al. 2000).

In the exogenous studies, four of the six included studies (Bos et al. 2012a; Hermans et al. 2008; van Wingen et al. 2008; van Wingen et al. 2009) used faces as the test stimuli. The faces used were adult, male or female, and angry or fearful. Control stimuli in these tasks were either happy or neutral faces, or abstract shapes with the scrambled tones of the faces. Of the two studies (Bos et al. 2010; Hermans et al. 2010) that did not use face stimuli, one used the sound of a baby crying, the other used a monetary incentive delay task. See Table 1 for more information.

**Table 1:** fMRI studies included in ALE (*n* = 6). Acute testosterone administration vs. placebo experiments in healthy participants using affective stimuli

Publication	Stimulus	N (mean age)	Foci (activation/deactivation)	roi/wb
Bos et al. (2010)	Crying infant sound	16 (20.8)	4(4/0)	roi and wb
Bos et al. (2012a)	Happy and fearful faces	12 (20.4)	1(1/0)	roi
Hermans et al. 2008)	Angry and happy faces	12 (22.6)	5(5/0)	wb and roi
Hermans et al. (2010)	Monetary incentive delay	12 (20.4)	3(2/1)	roi
van Wingen et al. (2008)	Faces of men/women with neutral affect	25 (42)	5(5/0)	roi and wb
van Wingen et al. (2009)	Angry and fearful faces	25 (42 and 23; two groups)	12(8/4)	wb and roi

In the endogenous tasks, four of the nine studies (Ackermann et al. 2012; Manuck et al. 2010; Stanton et al. 2009; Volman et al. 2011) used emotive faces as their test stimuli, in the same fashion as described in the previous paragraph. The other five studies used verbal insults (Denson et al. 2013), electric shocks (Choi et al. 2011), thermal pain (Vincent et al. 2013), videos of the participants’ own, and other, babies (Kuo et al. 2012), and the ultimatum game (Mehta and Beer 2010). See Table 2 for more information.

**Table 2:** fMRI studies included in ALE (*n* = 9). Experiments in healthy participants using affective stimuli, measuring endogenous testosterone levels

Publication	Stimulus	Control condition	Assay	N (mean age)	Foci (activation/deactivation)	roi/wb
Ackermann et al. (2012)	Neutral faces	Scrambled (M) and positive emotion faces (M + F)	Saliva	138F & 96M (22)	2(1/1)	roi
Choi et al. (2011)	Electric shocks, with placebo effect	Shocks without/low placebo	Blood	15M (25.3)	3(3/0)	roi
Denson et al. (2013)	Insults. Post provocation	Pre-provocation	Saliva	19M (22.6)	5(5/0)	wb and roi
Kuo et al. (2012)	Their babies	Other babies	Saliva	10M (34)	1(1/0)	roi
Manuck et al. (2010)	Angry and fearful faces	Shapes	Saliva	41M (45.6)	5(5/0)	roi
Mehta and Beer (2010)	Ultimatum game unfair offers	Fair offers	Saliva	15F & 17M; (23.3)	2(0/2)	roi
Stanton et al. (2009)	Angry faces	Neutral faces	Saliva	14F & 10M (21)	5 (1/4)	roi
Vincent et al. (2013)	Thermal pain (women on pill)	Thermal pain (women not on pill)	Blood	24F (not mentioned)	18(17/1) 4(3/1): *n* = 12	wb and roi
Volman et al. (2011)	Angry faces	Happy or neutral faces	Saliva	24M (19–28 range)	1(1/0)	voi

It is important to note that only tasks of an affective nature were included in this review. During the initial literature search a few papers were found to use cognitive measures, such as working memory and spatial awareness tasks, and they were excluded from the analyses. The range of affective tasks in the included studies can be considered rather wide, since they include gambling tasks, crying babies, angry faces, happy faces, etc. However there are not a sufficient number of studies focussing on each type of stimuli to conduct an analysis of each individually. Therefore we have grouped the tasks together in an attempt to ascertain neural regional activation that correlates with stimuli of a broadly affective nature. This should be kept in mind when discussing the results.

**Fig. 1:**
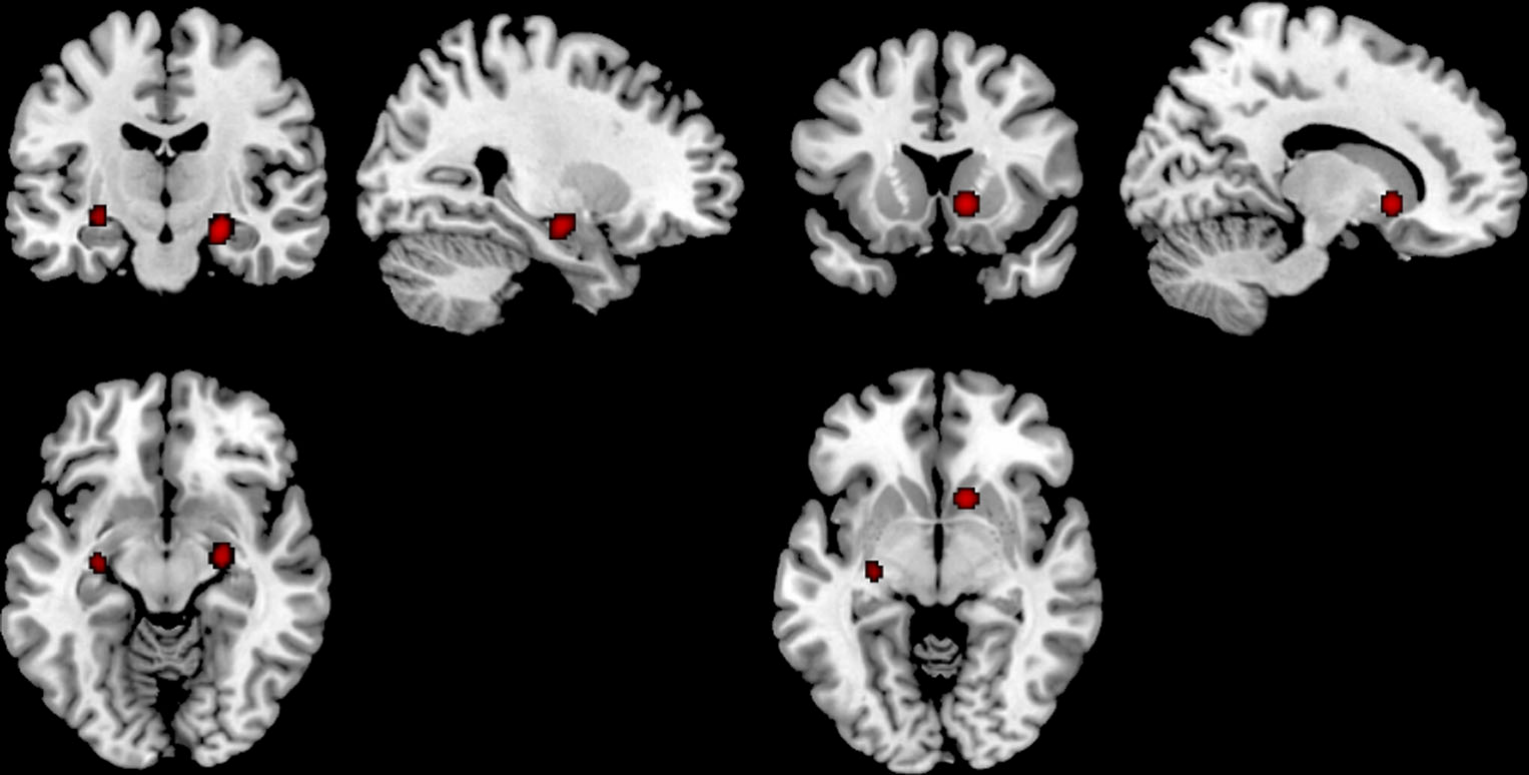
Activated regions in exogenous testosterone studies. 1) Left and right parahippocampal/amygdala regions and 2) Right caudate

**Fig. 2:**
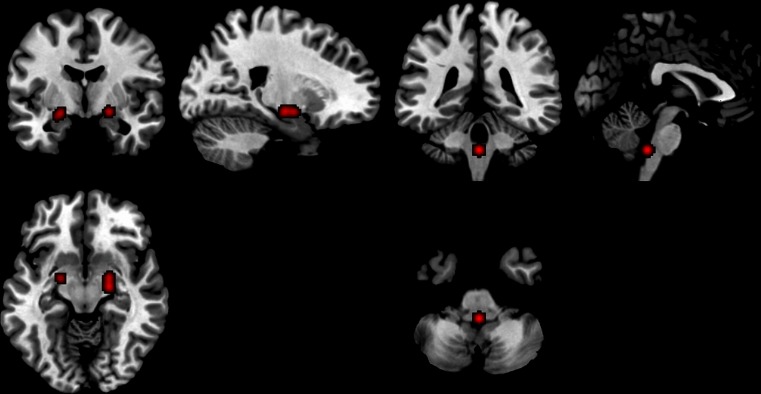
Activated regions in endogenous testosterone studies. 1) Right amygdala and left parahippocampal region and 2) Brain stem

**Fig. 3:**
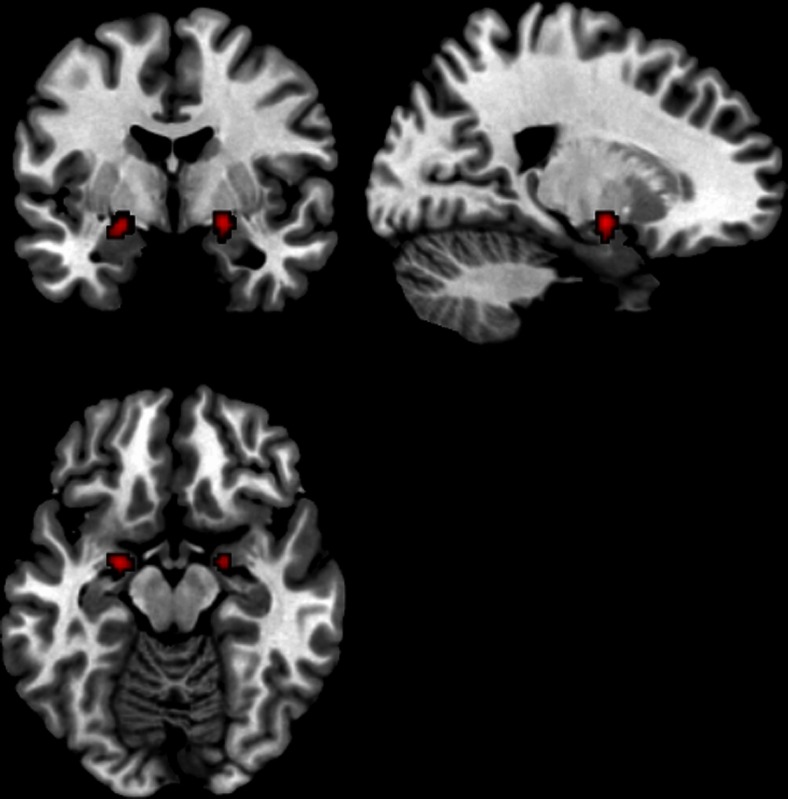
Activated regions in endogenous studies in men. Right and left parahippocampal/amygdala region

**Fig. 4:**
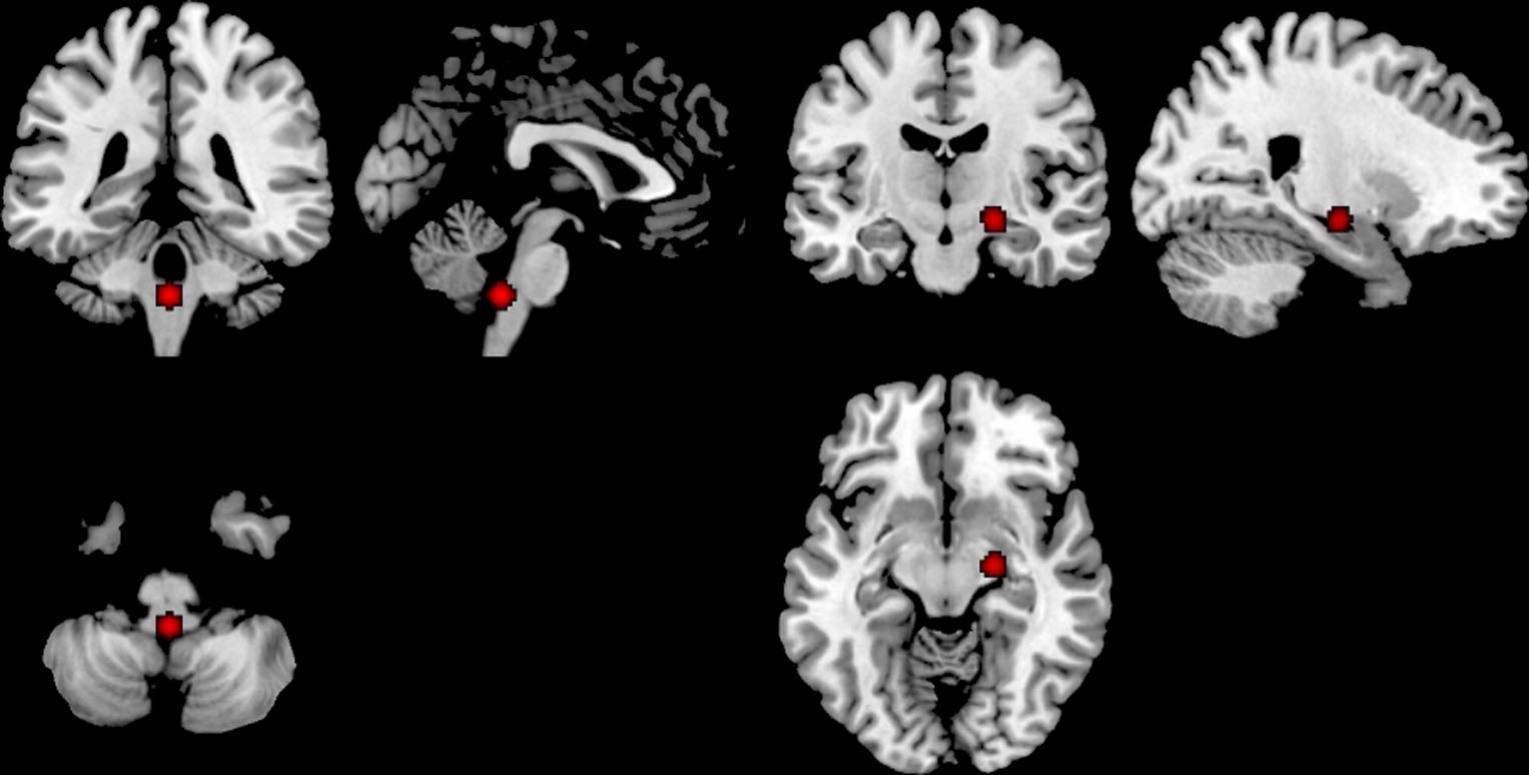
Activated regions in endogenous testosterone studies in women 1) Brain stem and 2) Right amygdala

**Fig. 5:**
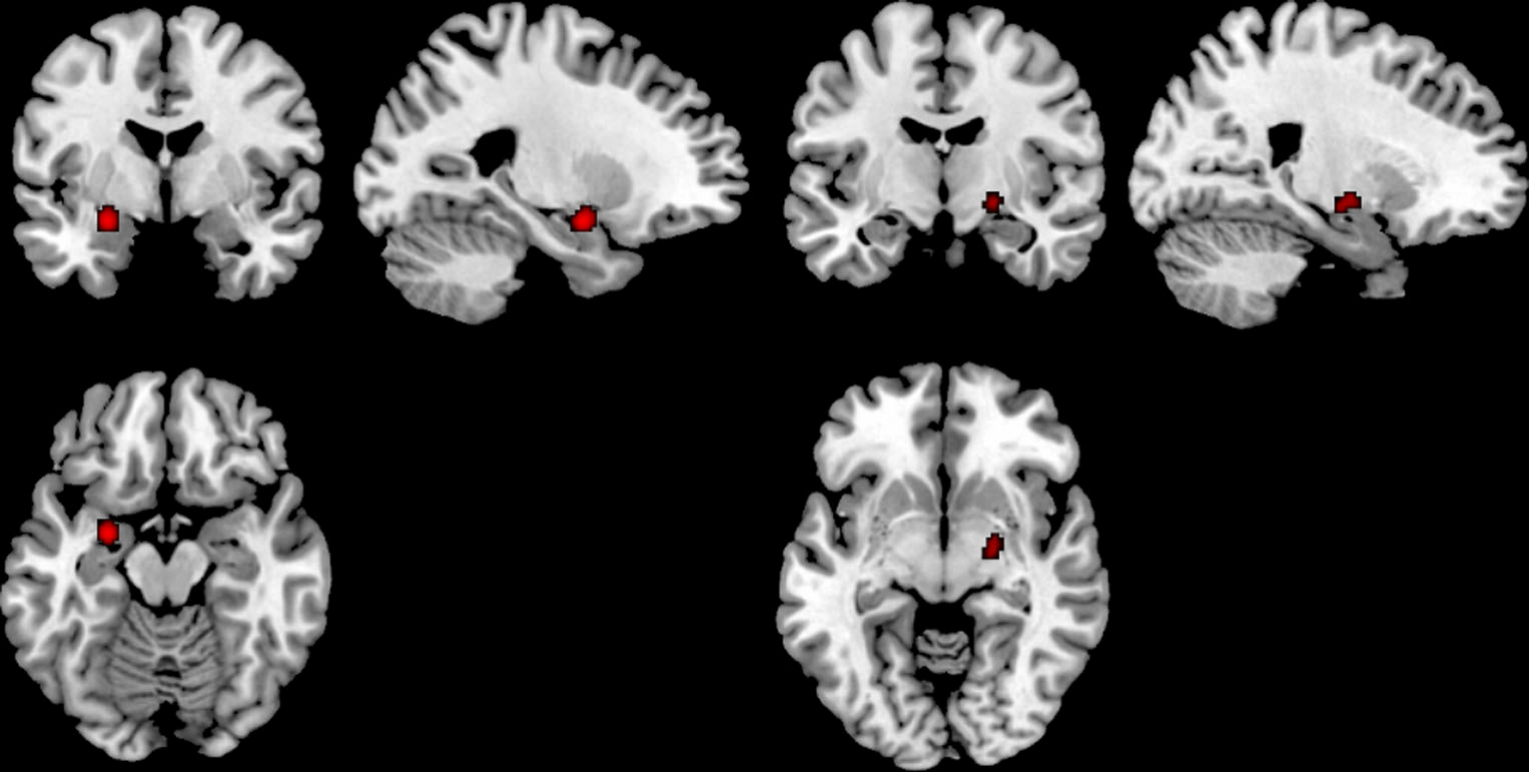
Deactivated regions in endogenous testosterone studies 1) Left parahippocampal region and 2) Right amygdala

### Activation likelihood estimation (ALE)

We used this statistical modelling technique, following the GingerALE protocol (http://www.brainmap.org/ale/) as devised by Eickhoff et al. (2012). ALE builds a 3-dimensional Gaussian kernel using foci coordinates from each included study to create a model activation (MA) map for each study. Between-study variance in the position of foci, possibly due to different templates used, or the differences between participants, are considered in the parameters of the kernel. This is done by weighting the foci reported by the sample size of each study. The MA maps for each study are combined for each separate meta-analysis, creating an experimental ALE map. This is tested against the null hypothesis that there is random variation in relation to the spatial orientation of neural activation for the specific meta-analysis, but that the within-study variation is fixed. A random effects model is employed by the ALE analysis technique, which assumes a higher than chance likelihood of consensus between different experiments, but not in relation to activation variance within each study. The null distribution map is permuted by the number of studies that constitute each meta-analysis. To correct for multiple comparisons, we used a threshold of *p* < 0.05 False Discovery Rate (FDR), and chose a minimum cluster size of 200 mm^3^ 134 in line with a recently published fMRI ALE meta-analysis (Brooks et al. 2013). We used an anatomical image overlay program called MRIcron to illustrate the results of our meta-analyses with Talairach coordinates. All of these steps combined help to control for publication bias with regard to reported foci. GingerALE considers a study to be “contributing”, if its coordinates are located within the boundaries of an ALE cluster, but does not discount other studies, which might be located near these boundaries but outside of the cluster, and may have also contributed to it.

### Structural VBM studies

The 16 VBM papers that were included in the meta-analysis, were heterogenous in terms of their sample, and also their study design. The populations differed in health/pathology, age, sex, and time of testosterone level assessment (sometimes many years before scanning, sometimes via amniocentesis). A number of these studies focussed on ROIs in the corpus callosum or corticospinal tract. Hence, these studies were discussed qualitatively in this paper, as we deemed it inappropriate to conduct an ALE analysis.

## Functional findings by MRI

### Activations in exogenous testosterone studies

Six studies with a total of 102 participants contributed 25 foci, 13 of which were ROI, to this analysis. Three significant peaks were found: Two foci contributed to the right caudate (12, 14, −4); three foci contributed to the right parahippocampal region (24, −12, −14); and two foci contributed to the left parahippocampal/amygdala region (−30, −14, −10). These activations were in response to the social-affective fMRI task stimuli in the testosterone >placebo condition (Fig. 1).

### Activations correlated to endogenous testosterone in men and women

Thirty one foci (14 ROIs) from eight studies (*n* = 227) were included. They showed three significant clusters correlating with testosterone levels, as activated by task stimuli: Five foci contributed to right amygdala (20, −12, −10); three foci contributed to left parahippocampal/amygdala region (−22, −4, −12); and three foci contributed to brainstem (0, −39, −43) (Fig. 2).

### Activations correlated to endogenous testosterone in men

Fifteen foci (11 of which were ROIs) from six experiments (*n* = 191) were included. Two clusters emerged as significantly activated, in interaction with testosterone levels, in response to task stimuli: The left parahippocampal/amygdala region, with three contributing foci, (−22, −4, −12); and the right parahippocampal/amygdala region, with two contributing foci, (20, −4, −10) (Fig. 3).

### Activations correlated to endogenous testosterone in women

Seventeen foci (4 of which were ROIs) from two studies (*n* = 36) were included. Two clusters were significantly activated, in interaction with testosterone levels, in response to task stimuli: Three foci contributed to the brain stem (0, −39, −43); and three foci contributed to the right amygdala (20, −12, −10) (Fig. 4).

### Deactivations correlated to endogenous testosterone in men and women

Negative correlations with activations were included in the deactivations analysis. Five studies (*n* = 308) of healthy men and women, including nine foci, all of which were ROIs, comprised the analysis. Two significant foci were found: Left parahippocampal/amygdala region (−26, −2, −14), with two contributing foci; and the right amygdala (20, −10, −8), with two contributing foci (Fig. 5).

### A closer look at activations and deactivations in endogenous testosterone studies

Our main ALE analyses reported that the left and right parahippocampal/amygdala regions were both significantly activated and deactivated during affective tasks. Therefore, we hypothesised that there might be functional differences in subregions of the parahippocampal/amygdala complex (see Terburg and van Honk 2013). Using the anatomy toolbox for SPM (Eickhoff et al. 2005), we examined coordinates obtained from studies included in our meta-analysis to gain a more accurate label for our ALE observations.

Specifically, activations related to endogenous levels of testosterone were in the right cornu ammonis (CA) hippocampus, and the left basolateral amygdala (BLA). Whereas, the deactivations we observed were located in the right hippocampal amygdaloid transition area (HATA), and the left superficial amygdala (SFA). Activation in the right CA hippocampus was in relation to two tasks: one in which men were told to recognise and match either fearful or angry faces, with scrambled images as a control (Manuck et al. 2010); and one that involved thermal pain (Vincent et al. 2013), during which women felt heat pain on their inner arm. In terms of left BLA activation, two tasks contributed: The angry/fearful faces task as mentioned above (Manuck et al. 2010), and a face task with a neutral > scrambled contrast in a male sample (Ackermann et al. 2012). Deactivation in the right HATA, which incorporated deactivation in the neighbouring right pallidum, was reported in a sample of men that viewed angry vs. neutral faces (Stanton et al. 2009); and also in the thermal pain task in women (Vincent et al. 2013). Left deactivation was specific to the SFA, with the contributing studies consisting of a task that contrasted angry vs. neutral faces in a male sample (Stanton et al. 2009), and a task in which both men and women viewed positive vs. neutral faces (Ackermann et al. 2012).

## Qualitative review of structural VBM studies in adults

Lentini et al. (2013) found that testosterone levels positively correlated with grey matter volume in the parahippocampus, amygdala, insular cortex, and the occipital lobes in male and female adults. There was a tendency towards a negative correlation between testosterone levels and parietal grey matter. Witte et al. (2010) studied 34 healthy male and female participants and found an inverse correlation between testosterone levels and the opercular left inferior frontal gyrus, with testosterone explaining 2.2 % of the variance. Lessov-Schlaggar et al. (2005) tested midlife testosterone levels in men to predict later life (10–16 years later) brain volumes, and higher levels of testosterone were associated with increased frontal and parietal volumes in the men in old age. However, left occipital lobe matter was inversely related to middle age testosterone levels. In a study of 68 healthy males, Moffat et al. (1997) found that testosterone did not correlate with total brain volume, left hemisphere volume, or right hemisphere volume. However, testosterone was positively correlated with the area of the posterior half of the corpus callosum. Kallai et al. (2005) found that testosterone levels in 40 women were not associated with lateral volumetric asymmetries in the hippocampus or amygdala, in a volumetrics study. Patwardhan et al. (2000) investigated three groups of men: Klinefelter’s patients on no testosterone supplements (*n* = 5), Klinefelter’s patients on testosterone supplementation for at least 5 years (*n* = 5), and healthy controls (*n* = 10). Those in the patients (no supplements) group showed reduced left and total temporal gray matter, and reduced total, left, and right superior temporal gyrus gray matter, which was no longer significant when total temporal volumes were controlled for.

In summary, adult testosterone levels are associated with larger limbic, insula and occipital lobe volumes, and smaller parietal lobe and opercular left inferior frontal gyrus volumes. Conversely, when levels of midlife testosterone in men are correlated with regional density in later life, larger gray matter volumes in frontal and parietal regions, and smaller occipital volumes are seen. Further, greater white matter volume in the posterior corpus collosum is associated with testosterone levels in men. There is some evidence to suggest that testosterone effects on brain volume are more pronounced in men than in women.

## Qualitative review of structural VBM studies in children

Two studies measured testosterone levels before birth, and linked these to brain volumes in childhood. Lombardo et al. (2012) used fetal testosterone (FT), gathered via amniocentesis in the second trimester, to predict gray matter volumes in typically developing 8–11 year old girls and boys. FT positively predicted volumes in the right temporo-parietal junction/posterior superior temporal sulcus, bilateral somatosensory, motor, and premotor cortices, and left ventromedial amygdala. FT was predicative of lower GM volumes along the bilateral sylvian fissures, specifically Heschl’s gyrus, posterior lateral orbitofrontal cortex, anterior insula, pars triangularis, and left middle and superior temporal gyrus. Chura et al. (2010) also tested FT, in 28 normally developing, right handed boys between 8 and 11 years of age. There were no relationships between FT and total grey matter or total white matter, or the volumes of sections of the study’s ROIs (sections of the corpus callosum). There was a significant positive relationship between FT and rightward (right > left) asymmetry of the isthmus section of the corpus callosum.

Other studies measured testosterone and its link to brain volume, not during fetal development, but rather during pre-adolescence and puberty. Neufang et al. (2009) tested 46 children aged between 8 and 15, assessing for correlations between grey matter volumes and circulating testosterone. Grey matter volume (GMV) of the amygdala correlated positively with testosterone, specifically in the older boys of the sample. The GMV of diencephalic structures such as the hypothalamus, mammillary bodies, and ventral thalamus were also positively associated with testosterone levels, with the effect being more significant in boys. Hippocampal GMV was negatively correlated with testosterone, as was left parietal cortex, including precuneus and superior parietal gyrus, with this effect seen particularly in boys. Peper et al. (2009a) found, in 37 boys and 41 girls between 10 and 15 years of age, that testosterone levels were not related to any regional gray or white matter volumes, but in boys global GMV was positively associated. There was a trend (*p* = 0.07) among the girls for testosterone to be negatively associated with gray matter volume in the right fusiform gyrus, the left inferior frontal gyrus, the left middle temporal gyrus. Bramen et al. (2012) studied 130 healthy adolescents and reported that testosterone positively correlated with age in boys, but not girls. In boys, there were no associations detected between GMV and testosterone. In girls, however, testosterone levels negatively correlated with volume of the right amygdala. Higher testosterone in girls was also associated with smaller bilateral cortical GMV. Herve et al. (2009) tested 409 adolescents (12–18) and found that, in boys, there was a positive association between bioavailable testosterone and apparent gray matter density of the putative coriticospinal tract (even when controlling for age), but no such association in girls. In a sample of 85 boys and girls, matched for sexual maturity, Bramen et al. (2011) found that, in boys, testosterone levels correlated positively with cortical thickness of the posterior occipital cortex (specifically, the right lingual gyrus), but that same region was negatively associated with testosterone in girls. In girls, testosterone also associated negatively with thickness in the right superior temporal gyrus.

Three studies examined conditions that are known to be associated with differing levels of testosterone. Mueller et al. (2011a) studied 10 boys with familial male precocious puberty (FMPP; a condition associated with early excess androgen secretion) and 21 controls, all of whom performed a spatial awareness test. There was no difference in performance between the two groups, however, performance correlated negatively with medial prefrontal cortex and cuneus GMV in the FMPP group. In controls, there were positive correlations between performance and GMV in the cuneus and the right middle-occipital gyrus. Mueller et al. (2011b) tested 13 young boys with FMPP and 39 controls. They found that the boys with FMPP had greater GMV in the bilateral parahippocampal, fusiform gyri, and putamen compared to controls. Controls had larger GMV in the precentral gyrus. Furthermore, in this study, free testosterone levels were measured only in the patient group and found to be in the normal range of the age of their bones and tanner stages. Within said patient group, an inverse correlation between testosterone level and GMV was found in the bilateral striatum. Peper et al. (2009b) tested the hypothesis that having a male co-twin would alter levels of testosterone exposure in utero, and that this would link to differences in brain volume at age 9. 119 individual 9 year old twins were tested, and it was found that those with a male co-twin had larger total brain and cerebellum volume than those with a female twin. This was no longer significant once birth weight was controlled for. The same paper reported that the effect was no longer seen in twins’ adulthood.

In summary, a fetal testosterone study showed positive correlations with temporal, sensorimotor, and amygdala volumes, and negatively correlated with areas along the bilateral sylvian fissures and temporal regions. In young boys, testosterone appears to be associated with larger volumes in midbrain regions associated with arousal and motivated behaviors, as well as visual areas and GMV. However, in girls a different pattern emerged in that testosterone is linked to reduced brain volume in many of these regions.

## Discussion

We conducted an ALE meta-analysis to examine fMRI results related to both exogenous and endogenous levels of testosterone in healthy populations. We also conducted a qualitative review of studies using VBM that examined how brain structure is associated to levels of testosterone. Our main findings were that activity and structure of the parahippocampal/amygdala region were related to differing levels of both endogenous and exogenous testosterone. The association with this brain region appears to be modulated by gender and stage of development (e.g. fetal testosterone exposure, hormone levels during adolescence and adulthood), and future research needs to explore these factors further. Additionally, testosterone’s associations with the parahippocampal/amygdala region appear to be specific to sub-regions, as opposed to global activation or deactivation per se. Testosterone also appears to relate to brain structural development, but the VBM studies that we reviewed here show modulatory effects by gender, age, and other circulating hormones, and so more research is needed to clarify this area.

In the ALE findings of the exogenous testosterone studies, in addition to the amygdaloid-parahippocampal region, the right caudate was significantly activated. The caudate has been identified as a region that is functionally influenced by levels of FT in response to emotional facial stimuli (Lombardo et al. 2012). The caudate also activates in response to social threat regardless of testosterone level (Beyer et al. 2013). A positive relationship between testosterone and amygdala activation in aggression is consistently shown (Batrinos 2012) with a previous meta-analysis of neuroimaging studies finding that the amygdala activates in response to all visual emotional stimuli, particularly faces (Sergerie et al. 2008). In a meta-analysis of 385 neuroimaging studies, (Costafreda et al. 2008) found that both negative and positive emotional, visual stimuli were associated with activation of the amygdala, particularly fear and disgust.

In a recent review it was also noted that the amygdala both activated and deactivated in response to similar affective stimuli (Höfer et al. 2013), however, this previous review did not measure activation in sub-regions of the hippocampal-amygdala region. Although we used the SPM anatomy toolbox to distinguish amygdaloid and hippocampal subregions, our ALE analysis did not have the sensitivity to distinguish between activation in the basolateral and central medial amygdala regions, which is an important factor when considering the effects of testosterone on the brain (Terburg and van Honk 2013). Although the orbitofrontal cortex did not survive our ALE analyses, it has been linked with both the amygdala and brainstem in a threat heightening and inhibiting network as a response to social threat (Terburg and van Honk 2013). Their paper describes how, in high threat situations, testosterone decreases coupling of the amygdala with the orbitofrontal region and increases coupling of the amygdala with the brainstem, which may allow for a more reactive response. There is disagreement about the clear distinction of a dominance or social aggression network in these frontal-limbic-brainstem regions (Panksepp and Biven 2012). However, recent neuroimaging and behavioral research indicates a connection between these regions in affective behaviors related to threat responsivity and dominance, in conjunction with accompanying differences in hormonal profile (Hermans et al. 2008; van der Westhuizen and Solms 2015). More research is needed to draw firm conclusions on whether the dominance neural network is distinct, or part of other core affective brain systems.

As mentioned in the Methods Section, affective tasks containing differing stimuli were grouped together in order to allow for analysis. The seemingly simultaneous activation and deactivation of the relevant regions may be due to this, and makes clear that the amygdaloid/parahippocampal region is implicated in responses to a range of affective stimuli.

Several limitations should be noted. Many of the included studies examined only regions of interest (ROI). The amygdala and other areas were selected as ROIs based either on significant clusters found in preliminary whole brain main effects analyses, or due to established links between these regions, hormones, and pain and threat inhibitory pathways (Schulkin et al. 1998). There are limitations of the VBM technique, as used by studies included in the qualitative portion of this paper. The VBM method uses a probability estimate of brain volume, rather than the actual volume, that one might find if doing a manual tracing. Brains are also fitted to global templates to acquire the estimates. This is problematic particularly when studying child samples. The ALE method of conducting meta-analyses also has shortcomings. While it regards numbers of significant study foci weighted by sample size, it does not consider degree of statistical significance, BOLD signal strength, and cluster sizes reported by each study. There was, at times, much heterogeneity in the included studies, making direct comparisons difficult. In an attempt to combat this, only healthy samples were included in the ALEs, although the qualitative review sections included some pathological samples, such as groups with low or excess androgen. Due to the different hormonal profiles of men and women, including non-overlapping ranges of healthy levels of testosterone, it is also not ideal to compare the two populations in a hormone study.

In summary, the amygdala and hippocampus are regions which are significantly pinpointed in many testosterone studies. A more detailed look at the sub regions of these anatomical structures reveals that different regions may be associated with varying effects. To view the amygdala, for example, as a single structure while not regarding the different functions of its sub regions would be to miss the nuanced effects seen in affective, reactive behaviors (Swanson and Petrovich 1998; Terburg and van Honk 2013). The range of conditions and findings in this review make it clear that, when assessing the effects of testosterone on neural activity, structure and behavior, many variables need to be considered in order to appreciate the subtleties of the effects. It is vital to consider, for example, male and female populations separately, given their non-overlapping ranges in normal testosterone levels, as well as the different levels of other hormones between men and women (e.g. estrogen, cortisol, etc.). Additionally, exposure to testosterone in the womb is an instrumental factor in determining how testosterone affects behavior and reactivity to altering hormone levels later in life. The developmental role of testosterone in the expression of androgen receptors, as well as the role of genotype, appears to strongly influence hormone sensitivity in adulthood. Thus, to effectively consider how testosterone influences brain function (particularly in hippocampal/amygdala regions) future research should take into account the influence of developmental stages, sex, gender, social environment, genetic profiles, and other circulating hormones. These influences may also affect the presentation of clinical symptoms such as anxiety, depression and aggressive behavior that may be associated with aberrant testosterone effects on the brain.

## Electronic supplementary material

Supplementary Fig. 1 (DOC 40 kb)
