# Supplementary material for: A quantitative and qualitative review of the effects of testosterone on the function and structure of the human social-emotional brain
Source: Metab Brain Dis. 2015 Jun 16;31:157–67. doi: 10.1007/s11011-015-9692-y (PMC4718938; doi:10.1007/s11011-015-9692-y)
Supplement: Supplementary file 1 — (DOC 40 kb) [file 11011_2015_9692_MOESM1_ESM.doc]

1527 records identified through database searching

132 full-text articles assessed for eligibility

31 studies included. 15 in the quantitative synthesis (meta-analysis), and 16 in a qualitative discussion

Abstracts screened for eligibility

1395 full-text articles excluded:

290: animal studies

4: drug trial

8: prenatal hormone exposure

105: tumor or cancer studies

136: reviews or meta analyses

244 case reports, or n<8

129: studies of reproductive disorders

38: non-English

21: studies of menopause

82: gonadal or pituitary disorders

125: musculoskeletal, fitness, and adiposity

7: used PET or DTI scans

16: EEG sleep studies

28: children studies

14: unavailable abstracts (12 of the 14 were from the 80s or earlier)

154: Other medical conditions

101 excluded

14: EMG or EEG papers

2: fMRI coupling only

5: fMRI cognitive tasks

80: Behavioural studies

**Identification**

**Screening**

**Eligibility**

**Included**
